# A comprehensive database of high-throughput sequencing-based RNA secondary structure probing data (Structure Surfer)

**DOI:** 10.1186/s12859-016-1071-0

**Published:** 2016-05-17

**Authors:** Nathan D. Berkowitz, Ian M. Silverman, Daniel M. Childress, Hilal Kazan, Li-San Wang, Brian D. Gregory

**Affiliations:** Department of Biology, University of Pennsylvania, 433 S. University Ave., Philadelphia, PA 19104 USA; Genomics and Computational Biology Graduate Group, Philadelphia, USA; Cell and Molecular Biology Graduate Group, Philadelphia, USA; Department of Pathology and Laboratory Medicine, Perelman School of Medicine, University of Pennsylvania, Philadelphia, PA 19104 USA; Department of Computer Engineering, Antalya International University, Antalya, Turkey; Institute on Aging, Baltimore, USA; Penn Center for Bioinformatics, Perelman School of Medicine, University of Pennsylvania, Philadelphia, PA 19104 USA

## Abstract

**Background:**

RNA molecules fold into complex three-dimensional shapes, guided by the pattern of hydrogen bonding between nucleotides. This pattern of base pairing, known as RNA secondary structure, is critical to their cellular function. Recently several diverse methods have been developed to assay RNA secondary structure on a transcriptome-wide scale using high-throughput sequencing. Each approach has its own strengths and caveats, however there is no widely available tool for visualizing and comparing the results from these varied methods.

**Methods:**

To address this, we have developed Structure Surfer, a database and visualization tool for inspecting RNA secondary structure in six transcriptome-wide data sets from human and mouse (http://tesla.pcbi.upenn.edu/strucuturesurfer/). The data sets were generated using four different high-throughput sequencing based methods. Each one was analyzed with a scoring pipeline specific to its experimental design. Users of Structure Surfer have the ability to query individual loci as well as detect trends across multiple sites.

**Results:**

Here, we describe the included data sets and their differences. We illustrate the database’s function by examining known structural elements and we explore example use cases in which combined data is used to detect structural trends.

**Conclusions:**

In total, Structure Surfer provides an easy-to-use database and visualization interface for allowing users to interrogate the currently available transcriptome-wide RNA secondary structure information for mammals.

**Electronic supplementary material:**

The online version of this article (doi:10.1186/s12859-016-1071-0) contains supplementary material, which is available to authorized users.

## Background

RNA molecules serve as both conveyors of genetic information and as molecular machines with specific structural and catalytic functions in the cell. The function and regulation of every RNA molecule depends on its specific secondary structure, the intricate pattern of hydrogen bonds between complementary ribonucleotides that forms in its specific cellular environment. For instance, the ribosome, the central enzymatic complex in protein translation, is the classic example of an RNA-based machine, and thus the structure of its RNA subunits (ribosomal RNAs (rRNAs)) has been carefully dissected using detailed analyses. However, thousands of other structural RNA elements and catalytic RNAs exist in the cell, and the resources required to study them in more detail are mostly unavailable for large-scale use by the broader research community.

Advances in high-throughput sequencing technologies have allowed a significant increase in technical development of methods for studying RNA secondary structure on a transcriptome-wide scale. This has led to a diverse collection of sequencing-based approaches available for interrogating RNA secondary structure, and thus there are a number of large-scale data sets that are currently publicly available ([3, 5, 14, 15, 17]; see Methods). There are important methodological differences between these high-throughput structure-probing techniques, but the unifying principle is that they involve treating RNA samples with a reagent that selectively reacts with nucleotides depending on their base pairing status and then interrogating the treated RNA by high-throughput sequencing.

There are two methods that take advantage of ribonuclease (RNase)-mediated cleavage of RNA bases that are either double- or single-stranded (ds- and ssRNase, respectively). The first example is Parallel Analysis of RNA Structures (PARS), which requires two high-throughput sequencing libraries per sample. One library is treated with the ssRNase-specific RNase S1, while the other involves cleavage by the dsRNase-specific RNase V1. Both RNase treatments are titrated for single hit kinetics, meaning that each RNA molecule is cleaved only once by the nuclease used for treatment and thus it is not fully digested. The resulting singly cleaved RNA ends are immediately used as the substrate for ligation of a 5′ adapter molecule as the first step in high-throughput sequencing library preparation. Sequencing libraries prepared in this way produce reads whose 5′ ends directly correspond to the site of nuclease cleavage. The structure of an RNA molecule can then be inferred from the relative number of RNase S1 (unpaired) and V1 (paired) cuts at each nucleotide position [3].

Similar reagents are used in ds/ssRNA sequencing (ds/ssRNA-seq) but to a different effect. As with PARS, each RNA sample is split into two aliquots, which are then treated with either an ssRNase (RNaseONE) or dsRNase (RNase V1). However, instead of utilizing single hit kinetics on the RNA samples, the nucleases are allowed to proceed to full digestion. The resulting RNase-resistant regions from each treatment are sequenced, and a structure score is then computed for each detectable nucleotide position by directly comparing the sequencing read coverage between the dsRNA- and ssRNA-seq libraries ([6]; see Methods).

Two other approaches whose data we curated (see Methods) have combined chemical probing of RNA secondary structure with high-throughput sequencing technologies. For these approaches, unpaired RNA bases are labeled with a small molecule that inhibits elongation by reverse transcriptase (RT) used for cDNA synthesis during sequencing library preparation. This block in RT elongation results in termination of the cDNA molecules at the sites of these modified single-stranded nucleotides. Therefore, the resulting sequencing reads have 5′ ends at the site that was labeled by addition of the chemical adducts.

DMS-seq is named for the labeling reagent that it employs, dimethyl sulfate (DMS). This small molecule labels unpaired adenosines and cytosines, but does not react efficiently with these nucleotides when they are base paired with another nucleotide [10, 11]. Unlike the nuclease-based methods, DMS-seq does not include a reagent that specifically labels paired nucleotides. Instead, it directly assesses unpaired bases by measuring the DMS reactivity of nucleotides in natively folded RNA molecules compared to a control library where purified, denatured RNAs are treated with DMS and used as substrates in sequencing library preparation [14]. Double-stranded RNA regions are then inferred based on absence of DMS-seq signal at those nucleotides.

The other chemical-based structure probing method is selective 2′-hydroxyl acylation analyzed by primer extension sequencing (SHAPE-seq) [2, 8, 9], which uses any of several reagents that selectively label the 2′ hydroxyl of unpaired nucleotides. Like DMS, this label causes RT to terminate due to the inhibition of its ability to elongate, which ultimately results in sequencing reads whose 5′ terminal nucleotide corresponds to the labeled position. The 5′ end read depth of each position in the treated library can then be compared to the corresponding read depth in an untreated DMSO control. This approach has recently been updated to allow higher resolution of RNA secondary structure, especially in mammalian transcriptomes. Specifically, the recently developed in vivo click SHAPE (icSHAPE) added an additional improvement to this general approach, in which the 2′ hydroxyl-labeling reagent also contains a biotin moiety, allowing enrichment of labeled RNA fragments in the final sequencing libraries [15].

Although these techniques have been used to generate large-scale, broadly useful RNA structure probing data, there is no available resource that provides convenient access to these important data sets. Furthermore, there is no easy way to directly compare the results from these disparate approaches. To address this gap, we have developed Structure Surfer, a database for exploring and comparing data generated by these new high-throughput structure-probing techniques (http://tesla.pcbi.upenn.edu/strucuturesurfer/). To do this, we have curated a comprehensive database of RNA secondary structure scores produced by the described experimental approaches. Structure Surfer allows users to query individual genomic loci of interest and visualize the local structural environment to directly compare the various methods. Additionally, we have included a tool for aggregating data across multiple genomic loci that allows users to query transcriptome-wide structural trends in a collection of regions of interest (e.g. all transcript start codons). In total, Structure Surfer provides an important and easy-to-use resource for querying and comparing the high-throughput RNA secondary structure probing data that is available for mammalian transcriptomes.

## Construction and content

### ds/ssRNA-seq

HEK293T cells were seeded in 15 cm standard Corning tissue culture treated culture dishes (Sigma, St Louis, MO), and grown to 90 % confluence (approximately 18 million cells) in DMEM media (Life Technologies, San Diego, CA) supplemented with L-glutamine, 4.5 g/L D-glucose, 10 % fetal bovine serum (FBS (Atlanta Biologics, Atlanta, GA)), and Pen/Strep (Fisher Scientific, Waltham, MA).

RNA was isolated using the Qiagen miRNeasy RNA isolation kit following the manufacturer’s protocol (Qiagen, Valencia, CA). Two aliquots of 50 μg were used to make two replicates each of dsRNA-seq and ssRNA-seq libraries. These two types of structure-specific libraries were constructed as previously described [5, 6].

### Data resources

We curated RNA secondary structure data from two published studies of the human transcriptome: DMS-seq [14] and PARS [17], as well as previously unpublished structure scores from our ds/ssRNA-seq data set for human HEK293T cells. Additionally, we compiled the scores from both in vitro and in vivo icSHAPE experiments in mouse [15]. The icSHAPE scores were reformatted and loaded directly into a mySQL database. For the other methods, we obtained the raw high-throughput sequencing reads and calculated the structure scores similarly to the published method specific to each one. All scoring functions are summarized below.

### Genome coverage

For DMS-seq, PARS, and ds/ssRNA-seq data sets, raw reads were trimmed using cutadapt [7]. This step removes any contaminating 3′ adapter sequences caused by inserts shorter than the sequencing read length. Trimmed and untrimmed reads were combined and mapped to the human genome using TopHat [16]. Reads that could not be trimmed or mapped were discarded. We allowed up to two mismatches per read and a maximum edit distance of two. We discarded reads that mapped to more than five locations. For DMS-seq and PARS data, we computed the read coverage at each position in the genome with bedtools [12] using only the 5′ most nucleotide of each read. When calculating coverage for ds/ssRNA-seq, the entire read was used.

### DMS-seq scores

DMS labeling of a nucleotide causes RT to stall during the cDNA synthesis step of RNA-seq library construction. Unstructured nucleotides, those that are not involved in base pairing, are more highly reactive with DMS and thus they are more likely to be the site of such a stall. Thus, the resulting RNA-seq reads from this type of high-throughput structure probing technique have 5′ ends corresponding to the reactive, unpaired position. However, DMS labeling is not the only possible explanation for positions with a high tendency to cause RT stalls. For this reason, DMS-seq scores are expressed as nucleotide reactivity compared to a denatured control. The signal at each position is calculated based on the normalized number of 5′ read ends mapping to that position in the native structure library compared to the control [14].

$$ {R}_i=\frac{D_i/{D}_{max}}{C_i/{C}_{max}} $$

The reactivity *R* for position *i* is computed by first dividing the 5′ read end coverage at that position, *D*_*i*_ by the maximum 5′ read end coverage in the library, *D*_*max*_. The resulting ratio is divided by *C*_*i*_, the 5′ end read coverage at position *i* in the denatured control library normalized to the maximum 5′ end read coverage of the control library, *C*_*max*_. This reactivity score represents the degree of over-representation of RT stops in the DMS treated library compared with the control. High scores indicate positions where RT stops were frequent suggesting an unpaired nucleotide labeled by DMS.

### icSHAPE scores

As with DMS-seq, icSHAPE scoring reflects the higher reactivity of unpaired nucleotides compared to nucleotides involved in pairing. Reactivity is calculated from the count of 5′ read ends covering each position. These counts are normalized to counts from a no-reagent background library and adjusted according to a background base density [15].

$$ {R}_i=\left({D}_i-{C}_i\right)/(B) $$

Reactivity *R* for position *i* is based on the 5′ read end coverage at that position, *D*_*i*_, minus the coverage in the DMSO treated control library, *C*_*i*_. The background base density profile for each transcript, *B*, is defined as the sequencing depth of each base in the DMSO library.

### PARS scores

PARS scores reflect the differential cleavage of paired and unpaired regions by ribonucleases. Unpaired regions are more cleaved by RNase S1 while paired regions are more cleaved by RNase V1. Both enzymes create RNA fragments with 5′ phosphate groups by cleaving in their respective preferred regions. These ends are directly ligated onto sequencing primers. After cDNA synthesis and sequencing, each read has a 5′ end corresponding to a cleavage site. Scores were calculated from the count of 5′ read ends covering each position in the two nuclease treated libraries. Each score is based on the log ratio of the two coverage scores. The generalized log ratio is calculated by adding one count per position to both the numerator and the denominator before calculating the log ratio. This allows scoring of positions with positive counts in one of the two input libraries but no counts in the other library. Such positions are of interest because there is evidence that they are in a particular structural state, but the standard log ratio for them is undefined. A 5′ nucleotide (nt) rolling average is applied for smoothing. Positions with no coverage in either library were omitted [17].

$$ {S}_i={ \log}_2\left({\displaystyle \sum_{j=i-2}^{j=i+2}\frac{V{1}_j+5}{5}}\right)-{ \log}_2\left({\displaystyle \sum_{j=i-2}^{j=i+2}\frac{S{1}_j+5}{5}}\right) $$

PARS structure score *S* for position *i* is the generalized log ratio of the normalized 5′ end coverage for that position in the RNase V1 library and the corresponding coverage in the RNase S1 library. For each position, this value is calculated across the surrounding 5 nt window.

### Ds/ssRNA-seq scores

Unlike scores from the other methods, ds/ssRNA-seq scoring takes into account all positions from each read rather than the 5′ end coverage only. It employs similar reagents to PARS, but uses a longer enzyme treatment resulting in more complete digestion of each enzyme’s preferred structure type. After cDNA synthesis and sequencing, reads represent regions that were protected from structure specific digestion. For each position the score is the generalized log ratio of the normalized counts in the two libraries [6].

$$ {S}_i=lo{g}_2\left( ON{E}_i+1/V{1}_i+1\right) $$

### Visualization

The database’s plotting tool is implemented using the Python package PyGal. For plotting purposes, scores are scaled and re-centered to reveal local structural patterns and to make the data sets visually comparable. For the same reason, DMS and icSHAPE scores, which represent nucleotide reactivity as opposed to degree of structure, are inverted when displayed such that high scores indicate evidence of paired nucleotides in all data sets. Raw scores are available for download alongside the plots.

### Availability of data and materials

All ssRNA- and dsRNA-seq data generated for this study from HEK293T cells were deposited in GEO under the accession GSE72681. The PARS, DMS-seq, and icSHAPE data were downloaded from GEO using the accession numbers GSE50676, GSE45803, and GSE60034, respectively. The complete Structure Surfer database is available as a MySQL dump file at PennBox, https://upenn.app.box.com/s/1kj2f1w994sp3jmaakqhy9cw2w11vajk. The Python search tool and database schema can be found at GitHub, https://github.com/nberkow/StructureSurfer. The structure score profiles for ~100 RBPs (as shown in Fig. 3) calculated by Structure Surfer are available for download at http://tesla.pcbi.upenn.edu/structuresurfer. No login is required to access these resources.

## Utility and discussion

### Database content

The database contains structure scores from four methods including six individual experiments across human and mouse (Additional file 1: Table S1). The score coverage varies greatly between methods. Despite having the lowest sequencing depth, the ds/ssRNA-seq experiment produces the greatest score density. However this is not surprising given that the method uses all nucleotides covered by each read to generate scores while all of the other methods use only a single nucleotide per read when calculating scores. The most sparse scores come from the human PARS data set which covers only ~1 megabase of the transcriptome.

PARS, DMS-seq, and icSHAPE all use a single base pair per read to calculate scores but the libraries used in the DMS experiment were sequenced to a higher depth which likely explains its greater score density (Additional file 2: Table S2). The two icSHAPE experiments, which were sequenced to the highest depth of all the data sets included, produced an intermediate number of scored positions indicating that each scored position represents a greater number of reads on average. Each of the different methodologies produces scores that follow a distinct distribution (Additional file 3: Figure S1) making it difficult to draw direct comparisons between them. These differences are likely due in part to differences in reagent kinetics. PARS and ds/ssRNA-seq, for example, employ similar reagents but PARS digests RNA very mildly resulting in single hit kinetics while ds/ssRNA-seq involves digesting regions of RNA to near completion. Other differences may arise from normalization strategy, as with the two nucleotide labeling techniques. DMS uses, as a normalization control, a denatured RNA sample, which is more highly reactive to DMS. In contrast, icSHAPE uses an RNA sample treated with solvent only, which reflects absence of icSHAPE reactivity. Structure Surfer addresses this by allowing users to focus on local structure patterns and transcriptome-wide structure trends.

### Structure examples

**Fig. 1:**
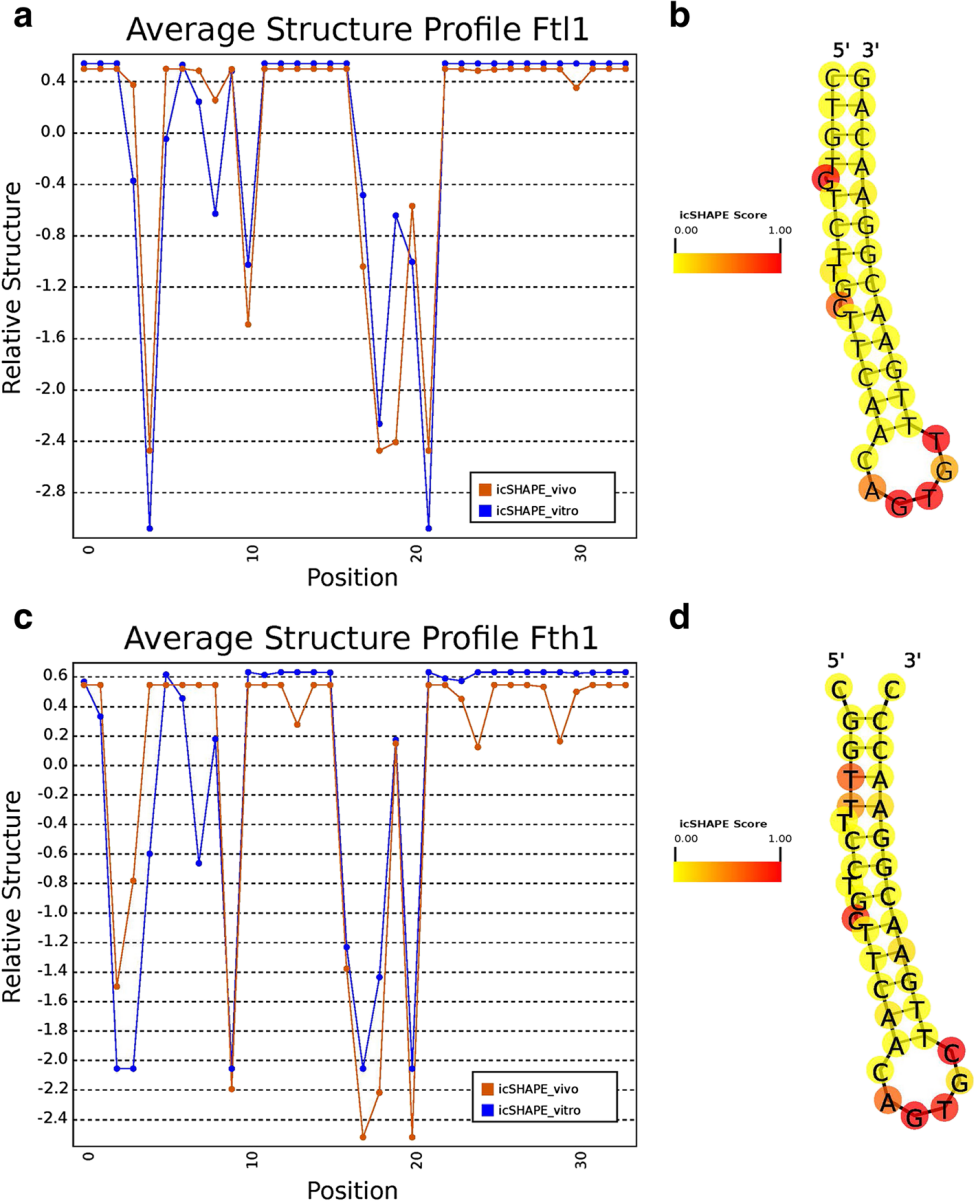
icSHAPE score profiles for the iron response element (IRE) hairpins of murine Ftl1 (**a**) and Fth1 (**c**) visualized using Structure Surfer’s standardized data output. The Ftl1 IRE is located at position 76 to 110 in the transcript and, in the genome, is located on chromosome seven from position 45459777 to 45459811 on the non-reference strand. Fth1’s IRE is located at position 83 to 117 in transcript variant 1. In the genome its coordinates are from 9982728 to 9982762 on the reference strand of chromosome 19. In vitro reactivity scores from the database are superimposed on *in silico* predicted structures for Ftl1 (**b**) and Fth1 (**d**) using SAVoR [4]. Red indicates positions with higher reactivity, showing evidence of low secondary structure. Positions colored in yellow have lower reactivity and are more likely paired

In order to develop our visualization of RNA secondary structure scores, we inspected a well-characterized class of highly structured elements, the iron response element (IRE). IREs are short stem-loops that act as binding sites for the RNA-binding protein (RBP) IRE-BP. They are found within the 5′ untranslated regions (UTRs) of several mRNAs including two that encode the heavy and light chains of Ferritin in mouse, *Fth1* and *Ftl1*, respectively. We visualized these two specific IREs using the database’s icSHAPE structure scores (Fig. 1a and c). In both structure score profiles, we see a five nucleotide stretch of low structure scores indicating an unpaired region. Indeed, each of these corresponds to the position of the unstructured loop region of the IRE. Also as expected, the structured stem region of the IRE has comparatively high scores. The 5′ and 3′ ends of the feature, which are not predicted to participate in the stem, have intermediate scores (Fig. 1a and c).

In both score profiles, there are several single nucleotide positions along the stem region with sharply low structure scores. We used the RNA annotation tool SAVoR [4] to superimpose icSHAPE reactivity scores onto RNAfold structures for the two loops (Fig. 1b, d, and Additional file 4: Figure S2). Because *Ftl1* is on the negative strand with respect to the genome, its scores were reversed in order before they were superimposed. Strikingly, the two most reactive positions outside of the loop region in *Ftl1* correspond to single nucleotide bulges in the stem at positions 5 and 11. This is not as clear in *Fth1*. While bulges in the predicted structure do generally correspond to peaks, as in the highly reactive bulge at position 11, there are two highly reactive nucleotides at positions 4 and 5, which are predicted to be paired. This provides evidence for the importance of structure probing techniques to define regions that differ in structure in vivo and *in silico*. One explanation for the differences is that *in silico* techniques do not always generate true RNA secondary structure. This may be a limitation of the algorithm used or it may be the result of nucleotide modification affecting structure in a way not reflected by the input sequence. For icSHAPE in particular, very high structure scores sometimes represent bases that are highly constrained, but in a way that makes them more rather than less reactive. More detailed experiments are needed to understand the exact source of disagreements between annotated structures and icSHAPE scores. Structure Surfer allows such differences to be detected easily and visually.

The human homologs of the mouse IRE features have no scores in any of the four human data sets, which illustrates a key issue to consider when dealing with RNA secondary structure. Structure measurement depends on RNA expression, sequencing depth, and technique specific biases. Many regions of potential interest have no scores or low score density. Fortunately, it is still possible to interrogate regions with low score density to detect overarching structure trends using a data aggregation approach.

**Fig. 2:**
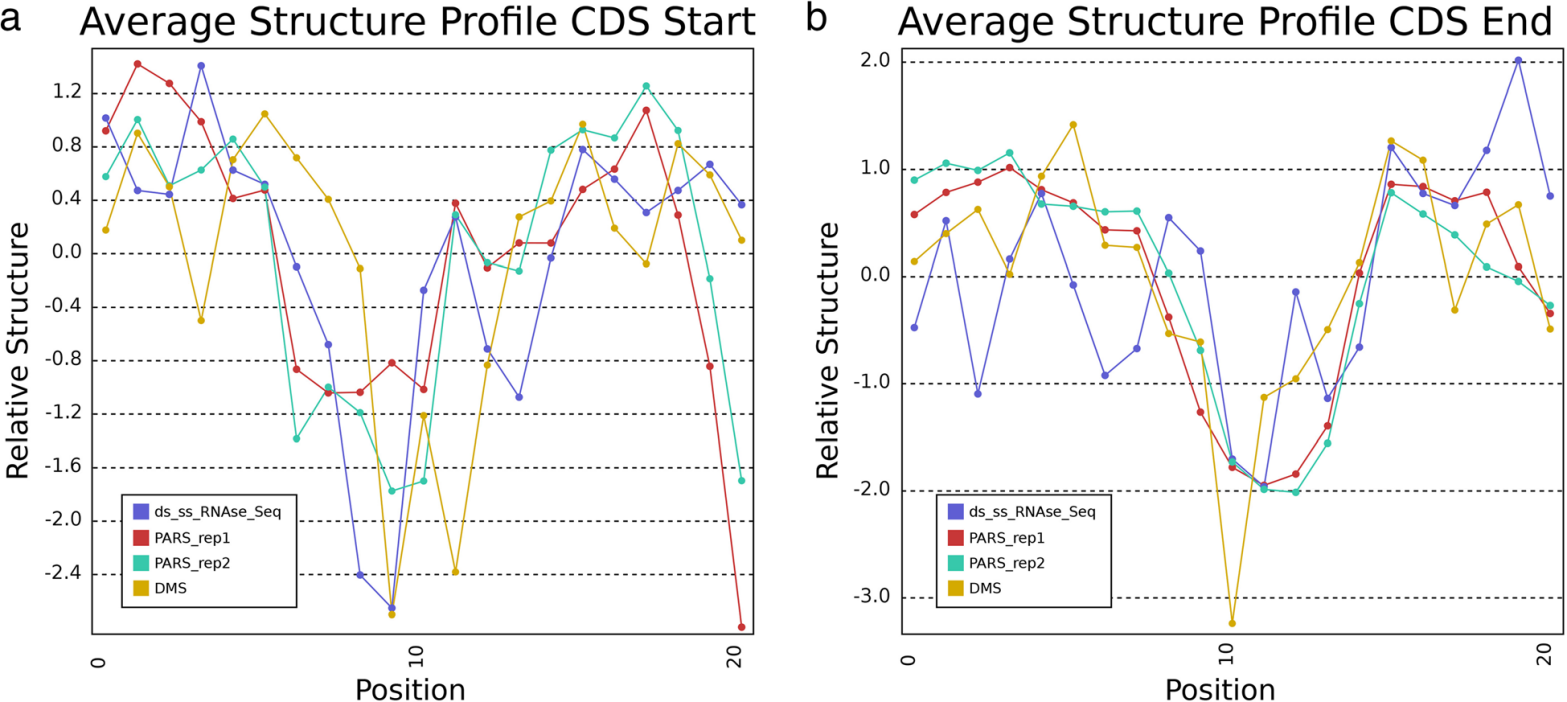
**a**-**b** Structure scores from the Structure Surfer database aggregated across all annotated human start codons (**a**) and stop codons (**b**). The three nucleotides of the start (ATG) and stop codons (e.g. TAA) occupy nucleotide positions 9–11 on each of the plots respectively. The score at each position is calculated as the average score across all nucleotides at that position relative to the codon. At both starts and stops, we note a dip in secondary structure consistent across experiments indicating that these positions, on average, have lower structure than the nucleotides surrounding them

Structure Surfer’s interface provides such an approach by allowing users to input multiple regions aggregated into a single bed file and find the average structure score for all of the incorporated data sets across this collection of regions. This is useful for investigating overall structural patterns across functionally related regions. For example, it has been noted that there are local decreases in RNA secondary structure at the start and stop of the coding sequence (CDS) [1, 5, 6, 17]. To test Structure Surfer’s aggregation mode, we queried the database with a set of sites containing every annotated CDS start codon in the human genome centered in a window of 9 nucleotides up- and downstream of these elements. Similarly, another file was entered using every CDS stop codon and their 9 nt up- and downstream surrounding sequences. When averaged across all of the input features, every human data set shows a dip in secondary structure around both the CDS start and stop codons (Fig. 2a and b, respectively). Individual CDS start and stop sites may have very low score densities, but taken together, their average scores indicate broad agreement between the data sets and agreement with this previously described structural trend in numerous eukaryotic organisms [1, 5, 6, 17]. This example shows how Structure Surfer can be used to reveal trends in RNA secondary structure across biologically related regions.

### Example use case: RNA-binding protein interaction motifs

As an example application of Structure Surfer, we also used it to query the structural patterns at and around RBP interacting motif sites. Many RBPs bind their target transcripts according to sequence specificity, however it is likely that the structural environment around these sequences is also important. A recent high-throughput study applied the RNAcompete protocol to identify sequence motifs for 244 RBPs across multiple organisms [13]. We selected the RBPs from human and mouse that were interrogated by this study, and scanned both genomes for matches to RNAcompete-derived motifs. For each selected RBP, we computed an average structure score across all matching sites (all data from these analyses can be downloaded from http://tesla.pcbi.upenn.edu/strucuturesurfer/RBP_motif_structure.pdf).

**Fig. 3:**
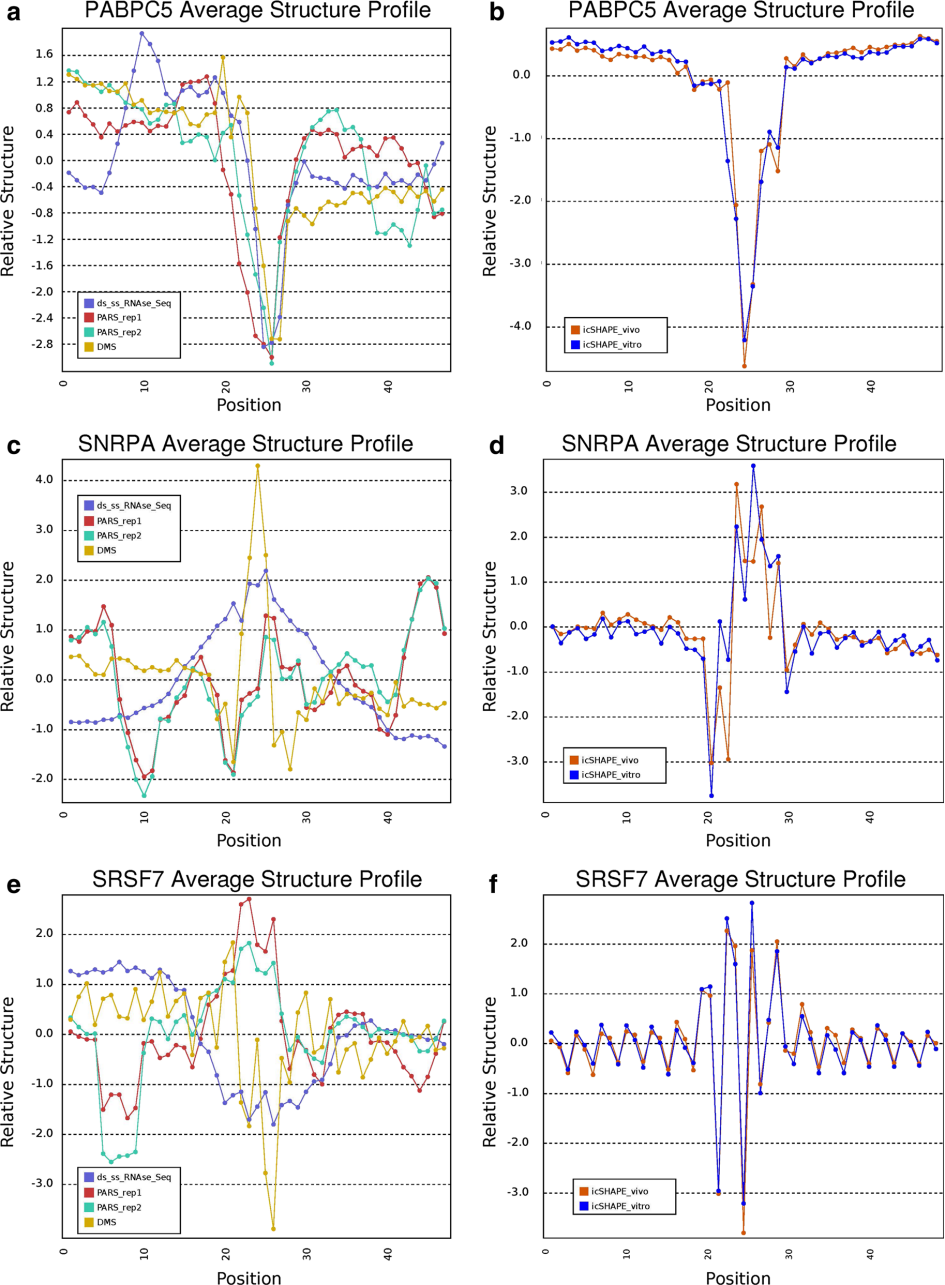
Examples of structure score aggregation using the data from Structure Surfer across RBP motif match sites for three RBPs, PABPC5 (**a**-**b**), SNRPA (**c**-**d**), and SRSF7 (**e**-**f**). Human structure scores are aggregated at match sites in the human exome (**a**, **c**, and **e**), and mouse scores are aggregated at match sites in the mouse exome (**b**, **d**, and **f**). In all examples, the RBP interacting motif sequence is a heptamer occupying nucleotide positions 21–27. The score at each position is calculated as the average score across all nucleotides at that position relative to the RBP motif. PABPC5 shows a consistent dip in secondary indicating that sites matching its motif have, on average, less secondary structure than surrounding nucleotides. The SNRPA motif shows the opposite trend. Specifically, the average structure scores at sites containing this motif are higher than the surrounding nucleotides indicating that these sites tend to be double stranded. Sites for SRSF7 show a more complex pattern in which the different experiments do not form a consensus. PARS demonstrates evidence for a peak in average secondary structure at SRSF7 motifs, while ds/ssRNA-seq and DMS display evidence for a dip in average secondary structure. The icSHAPE experiments both show a region where some positions appear to be involved in base pairing and others appear unpaired

We found several examples of RBPs whose predicted binding sites show a consensus structural environment across experiments. For example, motif matches for cytoplasmic polyadenine (polyA) binding protein 5 (PABPC5) show a strong unstructured trend when structure scores of all sites are averaged (Fig. 3a). We observe the same result when we search for PABPC5 sites in the mouse genome and average their icSHAPE scores (Fig. 3b). The opposite trend is found for motifs recognized by SNRPA, a component of the splicing machinery. All experiments report a local peak in structure at SNRPA motif sites in both human and mouse (Fig. 3c-d).

Unlike the examples above where we consistently find the same pattern across the different structure data sets, we also observe sites where there is not a consensus. For instance, the collection of predicted interaction sites of SRSF7 appear to be structured according to PARS, but unstructured according to DMS and ds/ssRNA-seq (Fig. 3e). Interestingly, the icSHAPE experiments report an average structural environment with some highly reactive positions and some positions that appear protected (Fig. 3f). One possible explanation for the icSHAPE result is that highly reactive sites compete for reagent with their slightly less reactive neighbors even if the entire region is unstructured. If this is the case it may also explain the difference in signal between the other methods. While it is difficult to interpret non-consensus sites, they may provide some insight into the types of features that are differentially detectable between the four methods.

## Conclusions

Structure Surfer is a database of RNA secondary structure information compiled from six different experiments across four distinct methods from human and mouse. The web interface allows users to visualize secondary structure patterns at any genomic region of interest. For instance, we visualized a known feature type, the IREs of murine Ferritin heavy and light chain mRNAs, and revealed a pattern of structure scores that match the *in silico* RNAfold-predicted secondary structure for these elements. When the scores provided by the structure probing methods are sparse, we find that a data aggregation approach reveals broad overall structural trends in a collection of transcript regions (i.e. the area around all transcript start codons). Therefore, we have also implemented a data aggregation option in the web interface to interrogate files containing a collection of such regions. Using this interface, we demonstrate the ability to visualize a known structural trend, specifically the dips in secondary structure at translation start and stop sites. Also using aggregation, we see intriguing patterns of secondary structure at predicted binding sites of specific RBPs. However, these are only two of the many possible use cases of Structure Surfer. Specifically, we hypothesize that there will be structural patterns corresponding to nuances in splicing, translation, and many other important processes.

## Declarations

### Ethics approval and consent to participate

Structure Surfer includes new and previously published data from mouse and human cell lines. Thus, this statement is not applicable to our study.

### Consent for publication

This is not applicable to this study.

### Availability and requirements

All ssRNA- and dsRNA-seq data generated for this study from HEK293T cells were deposited in GEO under the accession GSE72681. The PARS, DMS-seq, and icSHAPE data were downloaded from GEO using the accession numbers GSE50676, GSE45803, and GSE60034, respectively. The complete Structure Surfer database is available as a MySQL dump file at PennBox, https://upenn.app.box.com/s/1kj2f1w994sp3jmaakqhy9cw2w11vajk. The Python search tool and database schema can be found at GitHub, https://github.com/nberkow/StructureSurfer. The structure score profiles for ~100 RBPs (as shown in Fig. 3) calculated by Structure Surfer are available for download at http://tesla.pcbi.upenn.edu/structuresurfer. No login is required to access these resources.

## Additional files

Additional file 1: Table S1. The number of informative nucleotides in the data sets included in Structure Surfer. (DOCX 39 kb) Additional file 2: Table S2. The number of total reads in each data set analyzed for inclusion in Structure Surfer. (DOCX 59 kb) Additional file 3: Figure S1. Distinct score counts for the various data types of curated data from the high-throughput structure mapping approaches now available in Structure Surfer. Differences in method result in very different score distributions. (A) DMS scores show a distribution where low scores are common and extreme scores are rare. (B) The scores for ds/ssRNA-seq follow a broader distribution centered at zero. (C) Scores for icSHAPE show a more uniform distribution between zero and one. (D) PARS data sets are highly enriched for scores near zero, but more extreme scores are also present. (JPG 269 kb) Additional file 4: Figure S2. In vivo reactivity scores superimposed onto the IREs of mouse Ftl1 (A) and Fth1 (B). Red indicates positions with higher reactivity showing evidence of low secondary structure. Positions colored in yellow have lower reactivity and are more likely to be paired. (JPG 115 kb)

## Abbreviations

rRNAs: ribosomal RNAs
PARS: parallel analysis of RNA structures
RNase: ribonuclease
dsRNase: double-strand ribonuclease
ssRNase: single-stranded ribonuclease
dsRNA-seq: double-stranded RNA sequencing
ssRNA-seq: single-stranded RNA sequencing
RT: reverse transcriptase
DMS: dimethyl sulfate
DMS-seq: dimethyl sulfate sequencing
SHAPE-seq: selective 2′-hydroxyl acylation analyzed by primer extension sequencing
icSHAPE: in vivo click SHAPE
IRE: iron response element
RBP: RNA-binding protein
CDS: coding sequence
