# Supplementary material for: A comprehensive database of high-throughput sequencing-based RNA secondary structure probing data (Structure Surfer)
Source: BMC Bioinformatics. 2016 May 17;17:215. doi: 10.1186/s12859-016-1071-0 (PMC4869249; doi:10.1186/s12859-016-1071-0)
Supplement: Additional file 1: Table S1. — The number of informative nucleotides in the data sets included in Structure Surfer. (DOCX 39 kb) [file 12859_2016_1071_MOESM1_ESM.docx]

**Table S1: The number of informative nucleotides**

| **Experiment** | **Species** | **Scored Positions (M)** |
| --- | --- | --- |
| PARS replicate 1 | Human | 1.05 |
| PARS replicate 2 | Human | 0.81 |
| icSHAPE *in vitro* | Mouse | 14.65 |
| icSHAPE *in vivo* | Mouse | 18.53 |
| DMS | Human | 94.05 |
| ds_ss_RNA sequencing | Human | 282.52 |

**in the data sets included in Structure Surfer**
