# Supplementary material for: A comprehensive database of high-throughput sequencing-based RNA secondary structure probing data (Structure Surfer)
Source: BMC Bioinformatics. 2016 May 17;17:215. doi: 10.1186/s12859-016-1071-0 (PMC4869249; doi:10.1186/s12859-016-1071-0)
Supplement: Additional file 2: Table S2. — The number of total reads in each data set analyzed for inclusion in Structure Surfer. (DOCX 59 kb) [file 12859_2016_1071_MOESM2_ESM.docx]

**Table S2: The number of total reads in each data set analyzed for**

| **Library File** | **Raw Reads (M)** |
| --- | --- |
| DMS_K562denatured.fastq | 535.31 |
| DMS_k562vitro.fastq | 833.31 |
| PARS_rep1_s1.fastq | 156.49 |
| PARS_rep1_v1.fastq | 185.00 |
| PARS_rep2_s1.fastq | 186.24 |
| PARS_rep2_v2.fastq | 186.74 |
| v65 polyA(+) icSHAPE DMSO Biological Replicate 1 | 988.35 |
| v65 polyA(+) icSHAPE DMSO Biological Replicate 2 | 700.98 |
| v65 polyA(+) icSHAPE in vitro NAI-N3 Biological Replicate 1 | 781.94 |
| v65 polyA(+) icSHAPE in vitro NAI-N3 Biological Replicate 2 | 633.48 |
| v65 polyA(+) icSHAPE in vivo NAI-N3 Biological Replicate 1 | 298.36 |
| v65 polyA(+) icSHAPE in vivo NAI-N3 Biological Replicate 2 | 507.43 |
| HEK294T_invitro_dsRNA_seq.bam | 21.64 |
| HEK294T_invitro_ssRNA_seq.bam | 22.50 |

**inclusion in Structure Surfer**
