# Supplementary figures and images for: A comprehensive database of high-throughput sequencing-based RNA secondary structure probing data (Structure Surfer)
Source: BMC Bioinformatics. 2016 May 17;17:215. doi: 10.1186/s12859-016-1071-0 (PMC4869249; doi:10.1186/s12859-016-1071-0)

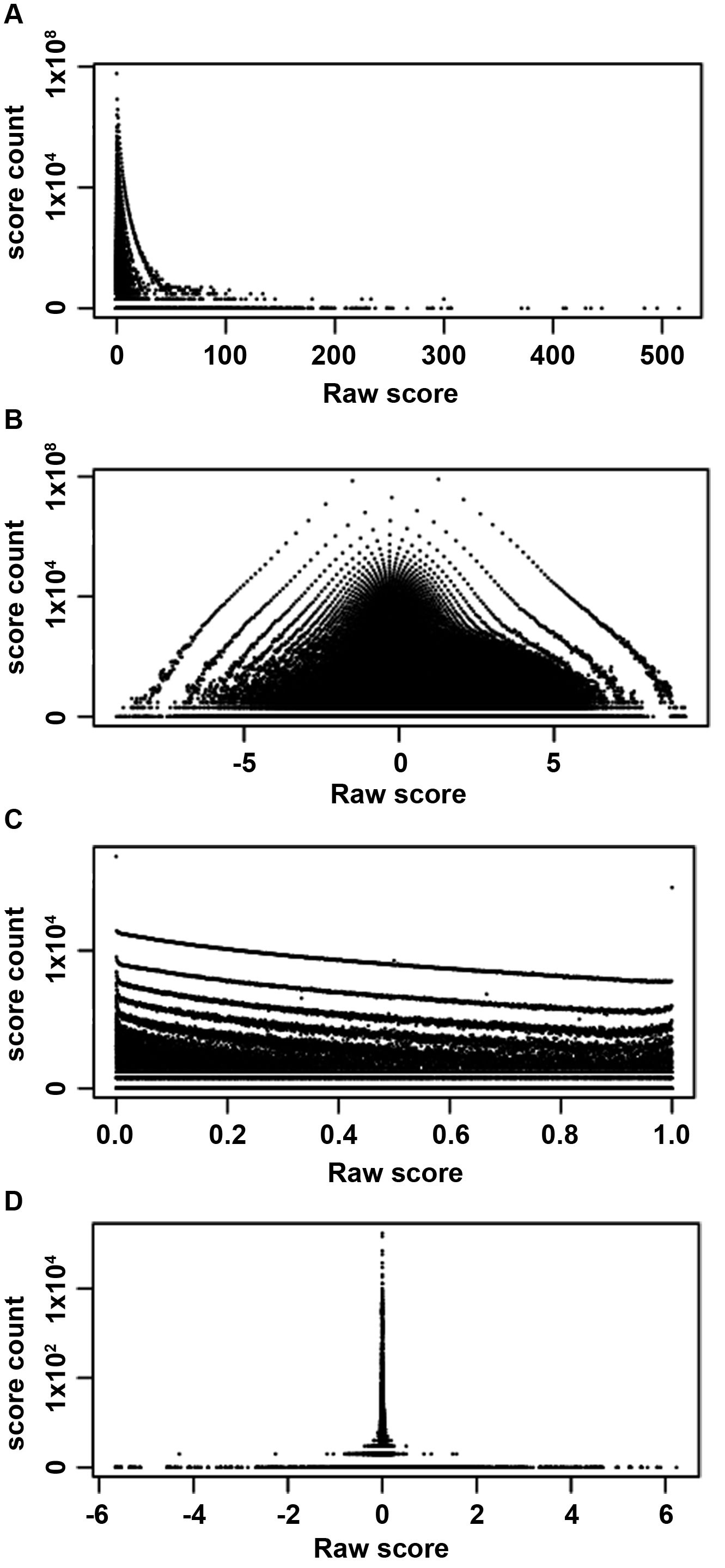

Supplement: Additional file 3: Figure S1. — Distinct score counts for the various data types of curated data from the high-throughput structure mapping approaches now available in Structure Surfer. Differences in method result in very different score distributions. (A) DMS scores show a distribution where low scores are common and extreme scores are rare. (B) The scores for ds/ssRNA-seq follow a broader distribution centered at zero. (C) Scores for icSHAPE show a more uniform distribution between zero and one. (D) PARS data sets are highly enriched for scores near zero, but more extreme scores are also present. (JPG 269 kb) [file 12859_2016_1071_MOESM3_ESM.jpg]

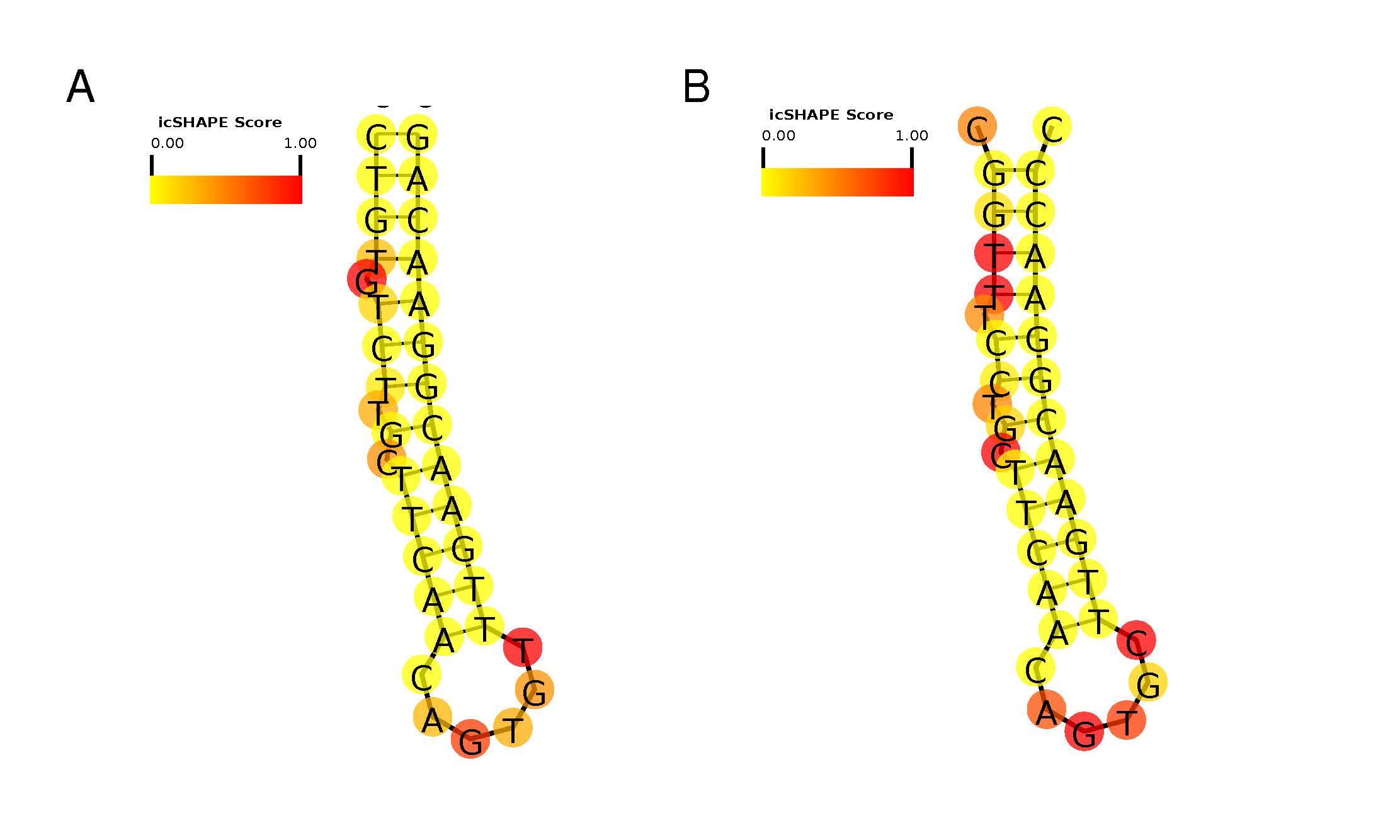

Supplement: Additional file 4: Figure S2. — In vivo reactivity scores superimposed onto the IREs of mouse Ftl1 (A) and Fth1 (B). Red indicates positions with higher reactivity showing evidence of low secondary structure. Positions colored in yellow have lower reactivity and are more likely to be paired. (JPG 115 kb) [file 12859_2016_1071_MOESM4_ESM.jpg]
